# Supplementary material for: The Health Literacy Status and Its Role in Interventions in Iran: A Systematic and Meta-Analysis
Source: Int J Environ Res Public Health. 2021 Apr 17;18(8):4260. doi: 10.3390/ijerph18084260 (PMC8073744; doi:10.3390/ijerph18084260)
Supplement: Supplementary file 1 [file ijerph-18-04260-s001.zip › ijerph-1135644-supplementary.pdf]

Supplementary File

Table S1. Inclusion and exclusion criteria.

	Inclusion Criteria	Exclusion Criteria
**Participants**	Iranian population (women and men) more than 18 years of age	Iranian women and men under 18 years of age and/ individuals with intellectual disabilities or mental illness
**Study Type**	Controlled trials, Quantitative designs, quasi-experimental, studies, and pre-test/post-test studies	Transversal descriptive studies
**Intervention**	All interventions designed based on HL strategies to improve/ facilitate functional, critical and interactive HL skills for Iranian population.<br><br>This includes patient and health providers coaching, patient prompts and skills workshops to improve individual's ability to promote healthy behaviors and health outcome.	HL was not specifically assessed Interventions; Specifically designed to improve HL; Interventions in mental health and oral health, school setting; Intervention protocols were not described
**Setting**	Any type of socio-health setting and clinical settings (e.g. primary care, secondary/tertiary care).	School setting; Mental health
**Outcomes**	- Health literacy skills<br>-Social and cognitive skills (self-efficacy, medication adherence, communication skills, self-care..)<br>- Knowledge of the disease/problem,<br>- Self-control/management of health problems:<br>- Prevention: preventive behaviors and knowledge participation in preventive measures<br>- Overall state of health: morbidity, mortality, quality of life, physiological indexes, etc.	
**Dissemination Type**	Scientific full text articles published in indexed scientific journals and conferences	Scientific articles before 2000.
**Language**	English/Persian	Articles written in languages other than English/Persian
**Others**	Published from ----<br>- Use of validated measures of HL and health outcome<br>- Assess relationship between HL and health outcome;<br>- Identifiable effect size	

Table S2. Joanna Briggs Institute critical appraisal checklist for analytical cross sectional studies.

	Critical appraisal checklist	Yes	No	Unclear	Not applicable
1	Were the criteria for inclusion in the sample clearly defined?	45	0	0	0
2	Was the exposure measure in valid and reliable way	42	0	3	0
3	Were objective, standard criteria used for measurement of the condition	45	0	0	0
4	Were confounding factors identified?	40	5	0	0
5	Were the study subject and the setting described in detail?	45	0	0	0
6	Were strategies to deal with confounding factor stated?	40	5	0	0
7	Was the outcome measured in a valid and reliable way?	45	0	0	0
8	Was appropriate statistical analysis used?	45	0	0	0

Table S3: Characteristics of the HL instrument

Measure	Description	Scoring	Internal consistency
TOFHLA (6,16)	It included 17 numerical skills items, 50 reading comprehension items	For TOFHLA, inadequate (0-59), marginal (60-74) and adequate (75-100).	For TOFLHLA: Cronbach's alpha is 79% for numerical skills and 0.88% for reading comprehension section.
STOFHLA (2,6)	STOFHLA: 4 numerical skills items and 36 reading comprehension item.	For STOFHLA, Inadequate (0-53 points), marginal (54-66 points) and adequate (67-100 points).	For S-TOFLHLA, Cronbach's alpha was 0.88 for numerical skills and 0.69 for reading comprehension section
REALM (7,8)	Consists of 66 items in area of medical words and health.	Inadequate (0-44), limited (45-60) and adequate (61-66).	Cronbach's alpha coefficients was 0.73.
NVS (9,59)	Consists of 6 items that require interpreting a nutrition label for a container of ice cream. The first four questions require numeracy skills and the last two require reading comprehension.	0–1 suggests high likelihood of limited HL; possible limited HL (2-3); and adequate HL (4-6)	Cronbach's alph was %0.69 for numerical skills and %0.78 for reading comprehension section
HELIA (64)	Consist of 33 items with answers according to a 5-point Liker. It loaded indicating a 5-factor solution for the questionnaire namely: access (6 items), reading (4 items), understanding (7 items), appraisal (4 items), and decision (12 items)	Inadequate (0-50), limited (50-66) and adequate (66-84), excellent (84-100)	Cronbach's alpha coefficients ranging from 0.72 to 0.89.

```
. metareg mean year ,wsse( sd )

Meta-regression                                     Number of obs  =       26
REML estimate of between-study variance             tau2           =    26.23
% residual variation due to heterogeneity           I-squared_res  =   10.24%
Proportion of between-study variance explained      Adj R-squared  =   70.88%
With Knapp-Hartung modification
```

mean	Coef.	Std. Err.	t	P>|t|	[95% Conf.	Interval]
year	5.087859	1.274795	3.99	0.001	2.456811	7.718907
_cons	-10197.44	2569.393	-3.97	0.001	-15500.41	-4894.474

Figure S1. Summary of meta-regression result for HL status in patient population.
